# The patient general satisfaction of mandibular single-implant overdentures and conventional complete dentures: Study protocol for a randomized crossover trial

**DOI:** 10.1097/MD.0000000000010721

**Published:** 2018-05-18

**Authors:** Manabu Kanazawa, Mariko Tanoue, Anna Miyayasu, Shin Takeshita, Daisuke Sato, Mari Asami, Thuy Vo Lam, Khaing Myat Thu, Ken Oda, Yuriko Komagamine, Shunsuke Minakuchi, Jocelyne Feine

**Affiliations:** a Gerodontology and Oral Rehabilitation, Graduate School of Medical and Dental Sciences, Tokyo Medical and Dental University; b Implant Center, Showa University Dental Hospital, Tokyo, Japan; c Oral Health and Society Division, Faculty of Dentistry, McGill University, Quebec, Canada.

**Keywords:** complete denture, cost-effectiveness, edentulous mandible, patient-reported outcome, randomized crossover trial, single-implant overdenture

## Abstract

**Background:**

Mandibular overdentures retained by a single implant placed in the midline of edentulous mandible have been reported to be more comfortable and function better than complete dentures. Although single-implant overdentures are still more costly than conventional complete dentures, there are a few studies which investigated whether mandibular single-implant overdentures are superior to complete dentures when patient general satisfaction is compared. The aim of this study is to assess patient general satisfaction with mandibular single-implant overdentures and complete dentures.

**Methods:**

This study is a randomized crossover trial to compare mandibular single-implant overdentures and complete dentures in edentulous individuals. Participant recruitment is ongoing at the time of this submission. Twenty-two participants will be recruited. New mandibular complete dentures will be fabricated. A single implant will be placed in the midline of the edentulous mandible. The mucosal surface of the complete denture around the implant will be relieved for 3 months. The participants will then be randomly allocated into 2 groups according to the order of the interventions; group 1 will receive single-implant overdentures first and will wear them for 2 months, followed by complete dentures for 2 months. Group 2 will receive the same treatments in a reverse order. After experiencing the 2 interventions, the participants will choose one of the mandibular prostheses, and yearly follow-up visits are planned for 5 years. The primary outcome of this trial is patient ratings of general satisfaction on 100 mm visual analog scales. Assessments of the prostheses and oral health-related quality of life will also be recorded as patient-reported outcomes. The secondary outcomes are cost and time for treatment. Masticatory efficiency and cognitive capacity will also be recorded. Furthermore, qualitative research will be performed to investigate the factors associated with success of these mandibular denture types. Clinical outcomes, such as implant survival rate, marginal bone loss, and prosthodontic complications, will also be recorded.

**Discussion:**

The results of this randomized crossover trial will clarify whether mandibular single implants and overdentures for edentulous individuals provide better patient general satisfaction when compared to conventional complete dentures.

**Trial registration:**

This clinical trial was registered at the University Hospital Medical Information Network (UMIN) Center (UMIN000017883).

## 1 Introduction

The age of populations continues to increase worldwide^[1]^; thus, the number of edentulous people is also increasing,^[2]^ along with their treatment needs. Conventional complete dentures (CDs) have been the traditional standard treatment option for edentulism. However, mandibular CDs often fail to satisfy denture wearers because of their mobility. Implant overdentures (IODs) are retained by implants and attachment systems under the denture base, and these have been reported to be a satisfactory treatment option for edentulous individuals. Furthermore, over the years there have been efforts to reduce the number of implants to retain IODs.^[3–5]^ These efforts were based on the need to reduce the cost and morbidity associated with use of multiple implants. Furthermore, 2 consensus statements were published, summarizing the evidence that 2 implants are adequate to retain a mandibular IOD.^[6,7]^

Efforts to reduce implant numbers are still ongoing, and several clinical studies on mandibular IODs retained by a single implant placed in the midline of the edentulous mandible (single-IOD [S-IOD]) were reported as having favorable outcomes.^[8–13]^ A recent study conducted by Kern et al^[14]^ reported that the survival rate of S-IOD with delayed loading protocol was up to 98% after 24 months of observation. Furthermore, based on a randomized controlled trial, Bryant et al^[15]^ reported that S-IOD had lower component costs, shorter treatment times and high patient satisfaction ratings when compared to 2-IOD. In addition, a systematic review^[16]^ revealed that S-IOD significantly decreased the marginal bone loss and number of implant failures compared to 2-IOD. Those reports indicated that an S-IOD can be considered as an alternative to 2-IOD for edentulous mandibles.

Although mandibular S-IODs are a less costly treatment option than 2-IODs, it is still more costly than a CD. Some edentulous patients may not be able to afford the costs of even a single implant and attachment system, as edentulous people tend to have low incomes.^[17]^ Cost is one of the main reasons that edentulous people refuse implant placement, despite their success.^[18]^ Especially in Japan, CDs are chosen as treatment modality for recovery of edentulousness because of a lot less expense and no invasiveness. However, patient-based outcomes such as patient general satisfaction should be assessed because patient-based outcomes are most appropriate variables, as these are based on the patients’ perception. A few published studies have shown whether mandibular S-IODs are superior to CDs in terms of general satisfaction. A cost-effectiveness analysis is necessary for proper assessment of these treatment options. Thus, the aim of this study is to determine the cost-effectiveness of mandibular S-IODs and CDs in a randomized crossover trial. The null hypothesis of this clinical trial is that there is no difference in general satisfaction with the prosthesis rated on 100 mm visual analogue scales (VAS) between mandibular S-IODs and CDs, considering treatment costs.

## 2 Methods/design

### 2.1 Trial design

This study was designed as a randomized crossover trial, comparing patient-reported outcomes of mandibular S-IODs with that of CDs. This study was registered with the University Hospital Medical Information Network (UMIN) Center UMIN000017883).

### 2.2 Participants

This study will be performed at the Dental Hospital, Tokyo Medical and Dental University, Japan. Recruitment and treatment protocol of the present study was approved by The Ethical Review Committee of the Faculty of Dentistry, the Tokyo Medical and Dental University (Register No. 1162). Eligibility criteria are as follows: edentulous mandible for at least 6 months at the time of implant placement, sufficient bone volume in the anterior region of the mandible, adequate understanding of written and spoken Japanese, and older than 50 years.

Exclusion criteria are as follows: uncontrolled systematic disease that could compromise implant surgery, current use of bisphosphonates, a history of chemotherapy, a history of radiotherapy in the head and neck region, heavy smoker, infectious disease, dementia, temporomandibular joint dysfunction, and orofacial pain.

### 2.3 Intervention

**Figure 1:**
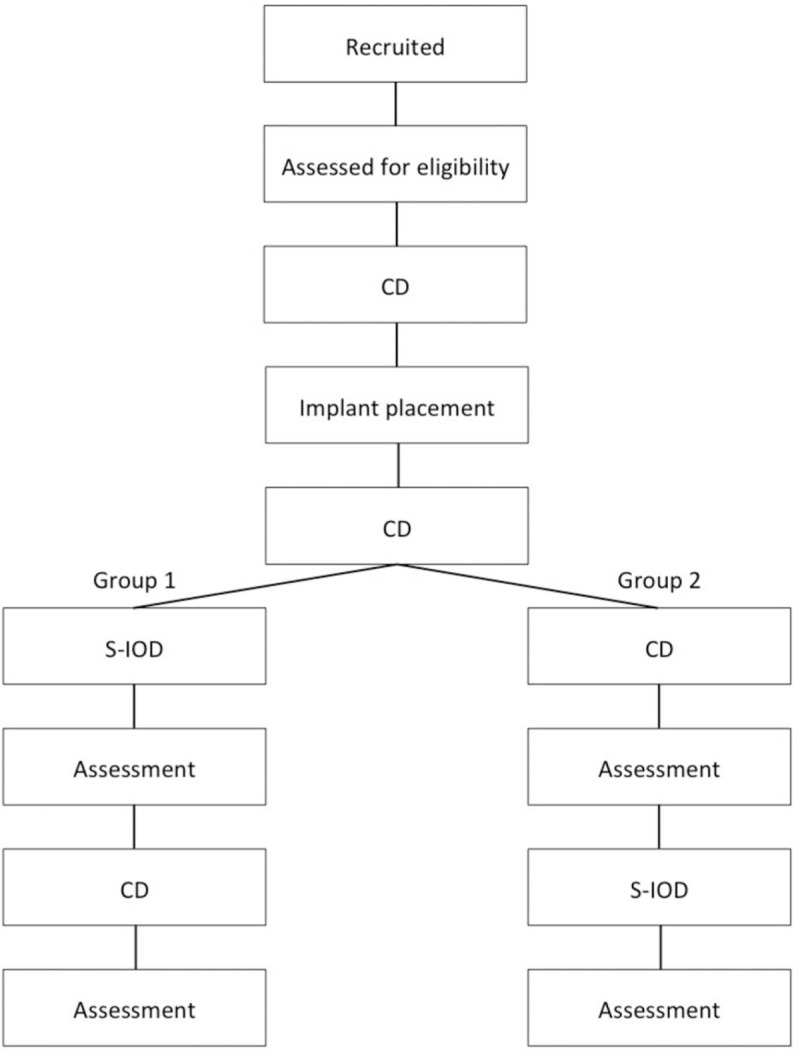
Flow diagram showing interventions. CD = complete denture.

The 2 interventions in this crossover trial are mandibular S-IODs and conventional CDs. Participants will be allocated into 2 groups. Group 1 will receive the S-IOD first, whereas group 2 will first receive the CD (Fig. 1).

Each participant will be screened to determine eligibility. A panoramic radiograph will be taken for each participant. New mandibular CDs with bilateral balanced occlusion will be fabricated for the participants who meet the eligibility criteria. Participants will be seen for follow-up appointments and necessary adjustments after denture delivery. All of the prosthodontic procedures will be performed by 1 experienced prosthodontist. After the participants adapt to their new mandibular CDs, they will be scheduled for implant surgery.

Preoperative planning of the implant surgery will be performed by a double scanning cone beam computed tomography (CT) technique (Finecube, Yoshida, Tokyo, Japan). The adjusted CDs will be used as a radiographic and surgical guide. Gutta-percha markers will be placed in the CD as the reference points. An initial CT scan will be performed for each of participants with their dentures seated in the mouth. The denture alone will then be scanned.^[19]^ Implant placement will be simulated using simulation software (NobelClinician, Nobelbiocare). The existing mandibular CD with a guide hole will be used as the surgical guide. Implant surgery will be performed under intravenous sedation. A minimum crestal incision will be used to elevate a full-thickness flap. One implant (SLA Ti BLT implant, 4.1 mm in diameter, 10 mm in length, Straumann) will be placed in the midline of the mandible, and a healing abutment (RC conical healing abutment, Straumann) will be connected to the implant. The upper face of the healing abutment will be same height as the mucosa. All of the surgical procedures will be performed by one experienced dental surgeon. To ensure osseointegration, the mucosal surface of the mandibular denture corresponding to the location of the healing abutment will be relieved for 3 months.

After 3 months, a locator abutment (Straumann) will be connected to the implant, and a locator cap (Straumann) will be incorporated into the mucosal surface of the CD for group 1. For group 2, the implant will remain covered by the healing cap, and the denture around the implant will again be relieved. The participants will wear their mandibular prostheses for 2 months, and then the prostheses will be changed. For group 1, the existing locator abutment will be exchanged for the healing abutment, and the denture base around the implant will be relieved. For group 2, the healing abutment will be changed for a locator abutment, and a locator cap will be incorporated into the mucosal surface of the CD. After 2 months, the participants will choose one of the mandibular prostheses, and yearly follow-up visits are planned for 5 years.

### 2.4 Outcomes

The primary outcome of this randomized crossover trial is patient satisfaction. Patient denture assessment (PDA) and oral health-related quality of life will also be evaluated as PRO. Ratings of satisfaction will be measured using 100 mm VAS. The general VAS question was stated as “How satisfied are you with your prosthesis?” PDA is an original questionnaire designed to measure the patients’ self-assessment of their dentures.^[20,21]^ Oral health-related quality of life will be measured using the Japanese version of the Oral Health Impact Profile for edentulous subjects (OHIP-EDENT-J) and the Japanese version of the General Oral Health Assessment Index (GOHAI). OHIP-EDENT-J is composed of 19 question items regarding 7 conceptual subscales; functional limitation, physical pain, psychological discomfort, physical disability, psychological disability, social disability, and handicap.^[22]^ The Japanese version of GOHAI contains 12 negatively worded questions.^[23]^ A food questionnaire and a brief self-administered diet history questionnaire ^[24]^ will also be completed. Assessment time will be as follows; before implant placement, before the crossover and after the first intervention and 2 months after the second intervention.

The participants will undergo the Japanese version of the Montreal Cognitive Assessment (MOCA-J). The MOCA-J is a brief detection tool for elderly people with mild cognitive impairment.^[25]^ The assessments will conducted at baseline and after both interventions.

The direct costs are the expenses associated with labor, equipment, and consumables. The labor costs will be determined by converting the time spent by the dentists and auxiliary/staff, etc for each patient. The indirect costs consist of patient's travelling expenses and time. The work time participants miss for each appointment will be included in the indirect costs.

Masticatory performance will be also estimated using color-changeable chewing gum (Xylitol Masticatory Performance Evaluating Gum XYLITOL, Lotte Co. Ltd., Tokyo, Japan),^[26]^ gummy jelly (UHA Mikakuto Co., Tokyo, Japan).^[27]^ Occlusal forces will be recorded using an occlusal force meter (Nagano Keiki, Tokyo, Japan). The clinical variables, such as implant survival rate, marginal bone loss, and prosthodontic complications, will be recorded annually up to 5 years.

Following all of the interventions, qualitative research will be performed by interviewing participants about problems associated with their mandibular S-IOD or CD. This qualitative research investigates factors associated with the patient-reported, as well as, the clinical outcomes.

### 2.5 Sample size

The sample size estimation was based on 100 mm VAS scores for patient's satisfaction. A between-group difference of 15 mm and expected standard deviations of 25 mm were sought in this study. Twenty participants are required for 80% power with a 2-sided alpha level of 0.05, based on the assumption that the VAS ratings are normally distributed. Taking into account the potential for drop-outs, 22 participants will be recruited.

### 2.6 Randomization

The participants will be randomly allocated into 2 treatment groups, namely group 1 and group 2, using sequentially numbered sealed opaque envelopes. Group 1 use S-IOD first, followed by CD. Group 2 is in the reverse order. Neither the participant nor the study team will know to which treatment group the participant has been assigned at the time of implant placement.

### 2.7 Blinding

The operator and the participants cannot be blinded. However, data entry and analysis personnel will be blinded to treatment assignment.

### 2.8 Statistical analysis

Statistical analyses will be performed for the outcomes to detect statistically significant differences between the 2 interventions, as well as within each group from baseline.

## 3 Discussion

The mandibular IOD was reported to be an effective treatment option for edentulous people. Several studies that aimed to reduce the number of implants to retain a mandibular IOD have been reported. Only one implant placed in the midline of the edentulous mandible is regarded as being adequate to retain an IOD. The mandibular S-IOD was reported to have favorable clinical results and high patient satisfaction ratings.^[8–12]^ Although implant placement is invasive and costly, and the S-IOD is still more expensive than a CD, some edentulous patients may complain about discomfort and functional limitations caused by retention and stability of mandibular CDs. There is still dearth of studies in a randomized control trial design about patient-based outcomes which compare S-IOD and CD. Therefore, this study aims to evaluate S-IODs and CDs in relation to patient general satisfaction.

It is true that the CDs in this study will cover the area under which implants are placed. However, the implants will be placed so that the upper face of the healing abutment will be at the same height as the mucosa, and the denture will be relieved around the mucosal surface covering the implant. Therefore, the condition of the CD in this study is similar to that of an actual CD. The protocol of this randomized clinical trial with a crossover design makes it possible for participants to try both the mandibular S-IOD and the CD. Furthermore, a crossover design requires fewer participants, reducing the time, effort, and cost for recruitment. On the contrary, the evaluation period for each mandibular prosthesis is 2 months because of its crossover design, which may be short to follow-up. That might be the limitation of the present study.

This clinical trial will clarify whether a single implant to retain a mandibular overdenture is a satisfactory treatment option compared to a conventional CD. These results will provide helpful information in choosing treatment options for edentulous patients

### 3.1 Trial status

At the time of manuscript submission (11 November 2017), patient recruitment is ongoing.

## Author contributions

ST conceived of and designed the study, will collect and analyze the data, drafted this manuscript. MK is the principal investigator who conceived the study and secured its funding, and who will perform all of the prosthodontic procedures. MT, AM, MA, TVL, KMT, KO and SM designed the study and are collecting and analyzing the data. YK drafted this manuscript. DS designed the study and will perform all surgical procedures. JF conceived of the study, will analyze and interpret the data, and assisting in the writing of this manuscript. All authors read and approved the final manuscript.

**Conceptualization:** Manabu Kanazawa, Shunsuke Minakuchi, Jocelune Feine.

**Data curation:** Mariko Tanoue, Anna Miyayasu, Shin Takeshita, Mari Asami, Thuy Vo Lam, Khaing Myat Thu, Ken Oda.

**Formal analysis:** Mariko Tanoue, Anna Miyayasu, Shin Takeshita, Mari Asami, Thuy Vo Lam, Khaing Myat Thu, Ken Oda.

**Funding acquisition:** Manabu Kanazawa, Shunsuke Minakuchi, Jocelune Feine.

**Investigation:** Mariko Tanoue, Anna Miyayasu, Shin Takeshita, Daisuke Sato, Mari Asami, Thuy Vo Lam, Khaing Myat Thu, Ken Oda.

**Methodology:** Manabu Kanazawa, Mariko Tanoue, Anna Miyayasu, Shin Takeshita, Daisuke Sato, Mari Asami, Thuy Vo Lam, Khaing Myat Thu, Ken Oda.

**Project administration:** Manabu Kanazawa.

**Resources:** Manabu Kanazawa.

**Supervision:** Manabu Kanazawa, Daisuke Sato, Shunsuke Minakuchi, Jocelune Feine.

**Validation:** Manabu Kanazawa.

**Writing – original draft:** Yuriko Komagamine.

**Writing – review and editing:** Manabu Kanazawa, Mariko Tanoue, Anna Miyayasu, Shin Takeshita, Daisuke Sato, Mari Asami, Thuy Vo Lam, Khaing Myat Thu, Ken Oda, Shunsuke Minakuchi, Jocelune Feine.
